# Deep Learning With Asymmetric Connections and Hebbian Updates

**DOI:** 10.3389/fncom.2019.00018

**Published:** 2019-04-04

**Authors:** Yali Amit

**Affiliations:** Department of Statistics, University of Chicago, Chicago, IL, United States

**Keywords:** Hebbian learning, asymmetric backpropagation, feedback connections, hinge loss, convolutional networks

## Abstract

We show that deep networks can be trained using Hebbian updates yielding similar performance to ordinary back-propagation on challenging image datasets. To overcome the unrealistic symmetry in connections between layers, implicit in back-propagation, the feedback weights are separate from the feedforward weights. The feedback weights are also updated with a local rule, the same as the feedforward weights—a weight is updated solely based on the product of activity of the units it connects. With fixed feedback weights as proposed in Lillicrap et al. (2016) performance degrades quickly as the depth of the network increases. If the feedforward and feedback weights are initialized with the same values, as proposed in Zipser and Rumelhart (1990), they remain the same throughout training thus precisely implementing back-propagation. We show that even when the weights are initialized differently and at random, and the algorithm is no longer performing back-propagation, performance is comparable on challenging datasets. We also propose a cost function whose derivative can be represented as a local Hebbian update on the last layer. Convolutional layers are updated with tied weights across space, which is not biologically plausible. We show that similar performance is achieved with untied layers, also known as locally connected layers, corresponding to the connectivity implied by the convolutional layers, but where weights are untied and updated separately. In the linear case we show theoretically that the convergence of the error to zero is accelerated by the update of the feedback weights.

## 1. Introduction

The success of multi-layer neural networks (deep networks) in a range of prediction tasks as well some observed similarities observed between the properties of the network units and cortical units (Yamins and DiCarlo, 2016), has raised the question of whether they can serve as models for processing in the cortex (Kriegeskorte, 2015; Marblestone et al., 2016). The feedforward architecture of these networks is clearly consistent with models of neural computation: a hierarchy of layers, where the units in each layer compute their activity in terms of the weighted sum of the units of the previous layer. The main challenge with respect to biological plausibility is in the way these networks are trained.

Training of feedforward networks is based on a loss function that compares the output of the top layer of the network to a target. Small random subsets of training data are then used to compute the gradient of the loss with respect to the weights of the network, and these are then updated by moving a small distance in the opposite direction of the gradient. Due to the particular structure of the function represented by these multi-layer networks the gradient is computed using back-propagation—an algorithmic formulation of the chain rule for differentiation (Rumelhart et al., 1986). In the feedforward step the input is passed *bottom-up* through the layers of the network to produce the output of the top layer and the loss is computed. Back-propagation proceeds top-down through the network. Successively two things occur in each layer: first, the unit activity in the layer is updated in terms of the layer above—feedback, then the weights feeding into this layer are updated. The gradient of each weight is a product of the activity of the units it connects—the feedforward pre-synaptic activity of the input unit in the lower layer and the feedback activity in the post-synaptic unit in the current layer. In that sense the gradient computation has the form of local Hebbian learning. However, a fundamental element of back-propagation is not biologically plausible as explained in Zipser and Rumelhart (1990) and Lillicrap et al. (2016). The feedback activity of a unit is computed as a function of the units in the layer above it in the hierarchy in terms of the *same* weight matrix used to compute the feedforward signal, implying a symmetric synaptic connectivity matrix.

Symmetry of weight connection is an unrealistic assumption. Although reciprocal physical connections between neurons are more common than would be expected at random, these connections are physically separated in entirely different regions of the neuron and can in no way be the same. The solution proposed both in Zipser and Rumelhart (1990) and in Lillicrap et al. (2016) is to create a separate system of feedback connections. The latter model is simpler in that the feedback connections are not updated so that the top-down feedback is always computed with the same weights. The earlier model proposes to update the feedback weights with the same increment as the feedforward weights, which as mentioned above has a Hebbian form. Assuming they are initialized with the same values, they will always have the same value. This guarantees that the back-propagation computation is executed by the network, but in effect reintroduces exact weight symmetry in the back-door, and is unrealistic. In contrast, the computation in Lillicrap et al. (2016) does not replicate back-propagation, as the feedback weights never change, but the price paid is that in deeper networks it performs quite poorly.

The main contribution of this paper is to experiment with the idea proposed in Zipser and Rumelhart (1990), but initialize the feedforward and feedback weights randomly (thus differently). We call this updated random feedback (URFB). We show that even though the feedback weights are never replicates of the feedforward weights, the network performance is comparable to back-propagation, even with deep networks on challenging benchmark datasets such as CIFAR10 and CIFAR100 (Krizhevsky et al., 2013). In contrast, the performance with fixed weights -fixed random feedback (FRFB), as in Lillicrap et al. (2016), degrades with depth. It was noted in Lillicrap et al. (2016) that in shallow networks the feedforward weights gradually align with the fixed feedback weights so that in the long run an approximate back-propagation is being computed, hence the name *feedback alignment*. We show in a number of experiments that this alignment phenomenon is much stronger in URFB even for deep networks. However, we also show that from the very initial iterations of the algorithm, long before the weights have aligned, the evolution of both the training and validation errors is comparable to that of back-propagation.

In our experiments we replace the commonly used unbounded rectified linear unit, with a saturated linearity σ(*x*) = min(max(*x*, −1), 1), which is more biologically plausible, as it is not unbounded, we avoid normalization layers whose gradient is quite complex and not easily amenable to neural computation, and we run all experiments with the simplest stochastic gradient descent that does not require any memory of earlier gradients. We also experiment with randomly zeroing out half of the connections, separately for feedforward and feedback connections. Thus, not only are the feedforward and feedback weights different, but connectivity is asymmetric. In a simplified setting we provide a mathematical argument for why the error decreases faster with updated feedback weights compared to fixed feedback weights.

Another issue arising in considering the biological plausibility of multilayer networks is how the teaching signal is incorporated in learning. The primary loss used for classification problems in the neural network literature is the cross-entropy of the target with respect to the *softmax* of the output layer (see section 3.2). The first step in back-propagation is computing the derivative of this loss with respect to the activities of the top layer. This derivative, which constitutes the feedback signal to the top layer, involves the computation of the softmax—a ratio of sums of exponentials of the activities of all the output units. It is not a local computation and is difficult to model with a neural network. As a second contribution we experiment with an alternative loss, motivated by the original perceptron loss, where the feedback signal is computed locally only in terms of the activity of the top-level unit and the correct target signal. It is based on the one-vs. all method commonly used with support vector machines in the multi-class setting and has been implemented through network models in Amit and Mascaro (2003), La Camera et al. (2004), and Amit and Walker (2012).

Finally, although convolutional layers are consistent with the structure of retinotopic layers in visual cortex, back-propagation through these layers is not biologically plausible. Since the weights of the filters applied across space are assumed identical, the gradient of the unique filter is computed as the sum of the gradients at each location. In the brain the connections corresponding to different spatial locations are physically different and one can't expect them to undergo coordinated updates, see Bartunov et al. (2018). This leads us to the final set of experiments where instead of purely convolutional layers we use a connectivity matrix that has the sparsity structure inherited from the convolution but the values in the matrix are “untied” and undergo independent local updates. Such layers are also called *locally connected* layers and have been used in Bartunov et al. (2018) in experiments with biologically plausible architectures. The memory requirements of such layers are much greater than for convolutional layers, as is the computation, so for these experiments we restrict to simpler architectures. Overall we observe the same phenomena as with convolutional layers, namely the update of the feedback connections yields performance close to that of regular back-propagation.

The paper is organized as follows. In section 2 we describe related work. In section 3 we describe the structure of a feedforward network, the back-propagation training algorithm and explain how it is modified with separate feedback weights. We describe the loss function and explain why it requires only local Hebbian type updates. In section 4 we report a number of experiments and illustrate some interesting properties of these networks. We show that performance of URFB is lower but close to back-propagation even in very deep networks, on more challenging data sets that actually require a deep network to achieve good results. We show that using locally-connected layers works, although not as well as convolutional networks, and that the resulting filters although not tied apriori show significant similarity across space. We illustrate the phenomenon of weight alignment that is much more pronounced in URFB. In section 5 we describe a simplified mathematical framework to study the properties of these algorithms and show some simulation results that verify that updating the feedback connections yields faster convergence than fixed feedback connections. We conclude with a discussion. Mathematical results and proofs are provided in the Appendix.

## 2. Related work

As indicated in the introduction, the issue of the weight symmetry required for feedback computation in back-propagation, was already raised by Zipser and Rumelhart (1990) and the idea of separating the feedback connections from the feedforward connections was proposed. They then suggested updating each feedforward connection and feedback connection with the same increment. Assuming all weights are initialized at the same value the resulting computation is equivalent to back-propagation. The problem is that this reintroduces the implausible symmetry since the feedback and feedforward weights end up being identical.

In Lillicrap et al. (2016) the simple idea of having fixed random feedback connections was explored and found to work well for shallow networks. However, the performance degrades as the depth of the network increases. It was noted that in shallow networks the feedforward weights gradually align with the fixed feedback weights so that in the long run an approximate back-propagation is being computed, hence the name *feedback alignment*. In Liao et al. (2016) the performance degradation of feedback alignment with depth was addressed by using layer-wise normalization of the outputs. This yielded results with fixed random feedback FRFB that are close to momentum based gradient descent of the back-propagation algorithm for certain network architectures. However, the propagation of the gradient through the normalization layer is complex and it is unclear how to implement it in a network. Furthermore Liao et al. (2016), showed that a simple transfer of information on the sign of the actual back-propagation gradient yields an improvement on using the purely random back-propagation matrix. It is however unclear how such information could be transmitted between different synapses.

In Whittington and Bogacz (2017) a model for training a multilayer network is proposed using a predictive coding framework. However it appears that the model assumes symmetric connections, i.e., the strength of the connection from an error node and a variable in the preceding layer is the same as the reverse connection. A similar issue arises in Roelfsema and Holtmaat (2018), where in the analysis of their algorithm, they assume that in the long run, since the updates are the same, the synaptic values are the same. This is approximately true, in the sense that the correlations between feedforward and feedback weights increase but significant improvement in error rates are observed even early on when the correlations are weak.

Burbank (2015) implements a proposal similar to Zipser and Rumelhart (1990) in the context of an autoencoder and attempts to find STDP rules that can implement the same increment for the feedforward and feedback connections. Again it is assumed that the initial conditions are very similar so that at each step the feedforward and feedback weights are closely aligned.

In a recently archived paper (Pozzi et al., 2018) also goes back to the proposal in Zipser and Rumelhart (1990). However, as in our paper, they experiment with *different* initializations of the feedforward and feedback connections. They introduce a pairing of feedback and feedforward units to model the gating of information from the feedforward pass and the feedback pass. Algorithmically, the only substantial difference to our proposal is in the error signal produced by the output layer, only connections to the output unit representing the correct class are updated.

Here we show that there is a natural way to update all units in the output layer so that subsequent synaptic modifications in the back-propagation are all Hebbian. The correct class unit is activated at the value 1 if the input is below a threshold, and the other classes are activated as −μ if the input is above a threshold. Thus, corrections occur through top-down feedback in the system when the inputs of any of the output units are not of sufficient magnitude and of the correct sign. We show that this approach works well even in much deeper networks with several convolutional layers and with more challenging data sets. We also present a mathematical analysis of the linearized version of this algorithm and show that the error converges faster when the feedback weights are updated compared to when they are held fixed as in Lillicrap et al. (2016).

Lee et al. (2015) and Bartunov et al. (2018) study target propagation where an error signal is computed in each hidden unit as the difference between the feedforward activity of that unit and a target value propagated from above with feedback connections that are separate from the feedforward connections. The feedback connections between each two consecutive layers are trained to approximate the inverse of the feedforward function between those layers, i.e., the non-linearity applied to the linear transformation of the lower layer. In Bartunov et al. (2018) they analyze the performance of this method on a number of image classification problems and use locally connected layers instead of convolutional layers. In target propagation the losses for both the forward and the backward connections rely on magnitudes of differences between signals requiring a more complex synaptic modification mechanism than simple products of activities of pre and post-synaptic neurons as proposed in our model.

Such synaptic modification mechanisms are studied in Guerguiev et al. (2017). A biological model for the neuronal units is presented that combines the feedforward and feedback signals within each neuron, and produces an error signal assuming fixed feedback weights as in Lillicrap et al. (2016). The idea is to divide the neuron into two separate compartments one computing feedforward signals and one computing feedback signals, with different phases of learning involving different combinations of these two signals. In addition to computing an error signal internally to the neuron this model avoids the need to compute signed errors, which imply negative as well as positive neuronal activity. However, this is done by assuming the neuron can internally compute the difference in average voltage between two time intervals. In Sacramento et al. (2018) this model is extended to include an inhibitory neuron attached to each hidden unit neuron with plastic synaptic connections to and from the hidden unit. They claim that this eliminates the need to compute the feedback error in separate phases form the feedforward error.

In our model we simply assume that once the feedforward phase is complete the feedback signal *replaces* the feedforward signal at a unit—at the proper timing—to allow for the proper update of the incoming feedforward and outgoing feedback synapses.

## 3. The Updated Random Feedback Algorithm

In this section we first describe the structure of a multilayer network, how the back-propagation algorithm works and how we modify it to avoid symmetric connections and maintain simple Hebbian updates to both feedforward and feedback connections. We then describe a loss function, whose derivatives can be computed locally, yielding a Hebbian input dependent update of the weights connecting to the final output layer.

### 3.1. Updated Asymmetric Feedback Connections

A multi-layer network is composed of a sequence of layers 0, …, *L*. The data at the input layer is denoted *x*_0_. Each layer is composed of *n*_*l*_ units. Let *W*_*l,ij*_ be the feedforward weight connecting unit *j* in layer *l* − 1 to unit *i* in layer *l*. Let *x*_*l*_, *l* = 1, …, *L* be the output of layer *l*, this is computed as

(1) $$\begin{array}{l} x_{l,i}=\sigma \left(h_{l,i}\right),\;h_{l,i}=\overset{n_{l-1}}{\underset{j=1}{\sum }}W_{l,ij}x_{l-1,j},i=1,\ldots ,n_{l}.\\ \text{or }h_{l}=W_{l}x_{l-1}, \end{array}$$

where σ is some form of non-linearity and *W*_*l*_ is the *n*_*l*_ × *n*_*l* − 1_ matrix of weights connecting layer *l*−1 to layer *l*. We denote *h*_*l,i*_ the input of unit *i* of layer *l*. For classification problems with *C* classes the top layer *L*, also called the output layer, has *C* units *x*_*L*,1_, …, *x*_*L,C*_. In this last layer no non-linearity is applied, i.e., *x*_*L,i*_ = *h*_*L,i*_. For given input *x*_0_ we can write $x_{L}=N\left(x_{0},W\right)$, where N represents the function computed through the multiple layers of the network with the set of weights W. The classifier is then defined as:

$$ĉ\left(x_{0}\right)={\text{argmax}}_{i}x_{L,i}={\text{argmax}}_{i}N\left(x_{0},W\right).$$

**Figure 1:**
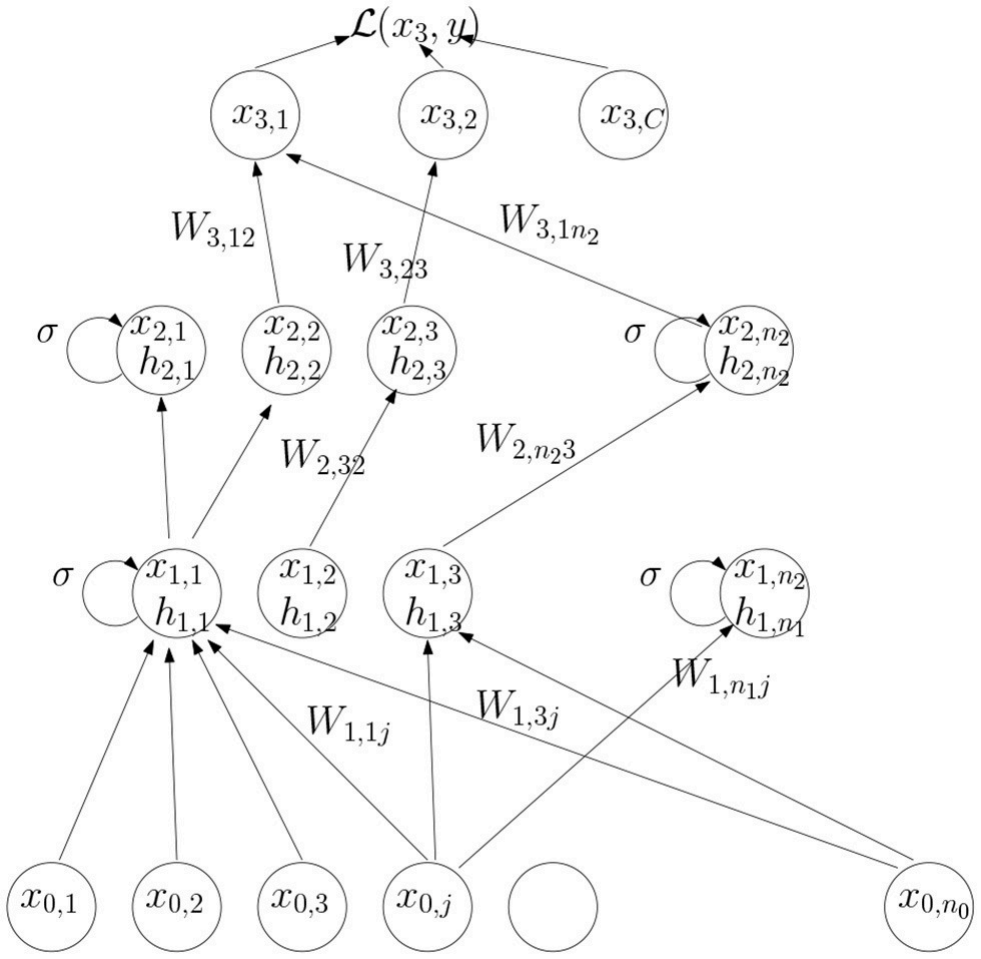
An illustration of the computations in a feedforward network.

A feedforward network with 3 layers is shown in Figure 1.

We define a loss $L\left(x_{L},y,W\right)$ comparing the activity of the output layer to a target value, an indicator vector denoting the correct class of the input. At each presentation of a training example the derivative $\partial L/\partial W_{l,ij}$ of the loss with respect to each weight is computed, and the value of the weight is updated as

$$W_{l,ij}=W_{l,ij}-\eta \partial L/\partial W_{l,ij},$$

where η is a small scalar called the time-step or learning rate. This is done in two phases. In the first phase, the feedforward phase, the input *x*_0_ is presented at layer *l* = 0 and passed successively through the layers *l* = 1, …, *L* as described in (1). In the second phase the derivatives are computed starting with *W*_*L,ij*_ for the top layer and successively moving down the hierarchy. At each layer the following two equalities hold due to the chain rule for differentiation:

$$\begin{array}{l} \frac{\partial L}{\partial W_{l,ij}}=\frac{\partial L}{\partial h_{l,i}}\frac{\partial h_{l,i}}{\partial W_{l,ij}}=\frac{\partial L}{\partial h_{l,i}}x_{l-1,j}\\ \frac{\partial L}{\partial h_{l,i}}=\sigma^{\prime }\left(h_{l,i}\right)\overset{n_{l+1}}{\underset{k=1}{\sum }}\frac{\partial L}{\partial h_{l+1,k}}W_{l+1,ki}. \end{array}$$

If we denote $\delta_{l,i}=\frac{\partial L}{\partial h_{l,i}}$ we can write this as:

(2) $$\begin{array}{l} \frac{\partial L}{\partial W_{l,ij}}=\delta_{l,i}x_{l-1,j}\\ \delta_{l,i}=\sigma^{\prime }\left(h_{l,i}\right)\overset{n_{l+1}}{\underset{k=1}{\sum }}\delta_{l+1,k}W_{l+1,ki}.\\ \text{or }\delta_{l}=\sigma^{\prime }\left(h_{l}\right)W_{l+1}^{t}\delta_{l+1}, \end{array}$$

where $\sigma^{\prime }\left(h_{l}\right)$ is the diagonal matrix with entries $\sigma^{\prime }\left(h_{l,i}\right)$ on the diagonal. So we see that the update to the synaptic weight *W*_*l,ij*_ is the product of the *feedback* activity at unit *i* of layer *l* denoted by δ_*l,i*_, also called the *error signal*, and the input activity from unit *j* of layer *l* − 1. The feedback activity (error signal) of layer *l* is computed in terms of the feedforward weights connecting unit *i* in layer *l* to all the units in layer *l* + 1. This is the symmetry problem.

We now separate the feedforward weights from the feedback weights. Let *R*_*l*+1, *ik*_ be the feedback weight connecting unit *k* of layer *l*+1 to unit *i* of layer *l*. The second equation in (2) becomes:

$$\delta_{l,i}=\sigma^{\prime }\left(h_{l,i}\right)\overset{n_{l+1}}{\underset{k=1}{\sum }}\delta_{l+1,k}R_{l+1,ik}.$$

**Figure 2:**
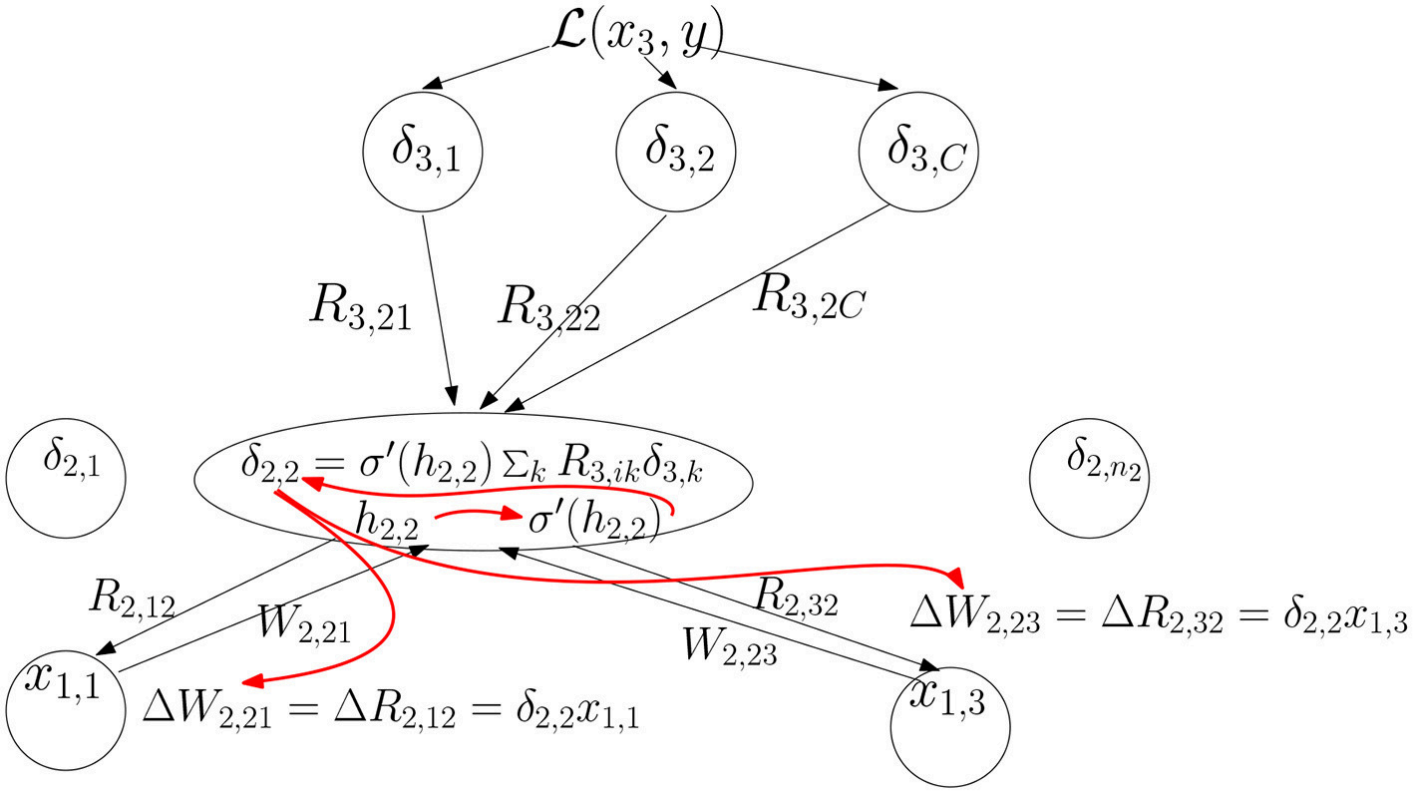
The feedback signals δ_3,*k*_ from layer 3 are combined linearly and then multiplied by $\sigma^{\prime }\left(h_{2,2}\right)$ to produce the feedback signal δ_2,2_. Then the update to the feedforward weights coming into unit (2, 2) and feedback weights coming out of that unit is computed. The red arrows indicate the order of computation.

If *R* = *W*^*t*^ we get the original back-propagation update. We illustrate the general updating scheme computation in Figure 2.

In Lillicrap et al. (2016) the values of *R* are held fixed at some random initial value, which we denote *fixed random feedback* (FRFB). In contrast, in our proposal, since *R*_*l*+1, *ik*_ connects the same units as *W*_*l*+1, *ki*_ it experiences the same pre and post-synaptic activity and so will be updated by the same Hebbian increment - δ_*l*+1, *k*_*x*_*l,i*_. We call this method *updated random feedback* - URFB. If the initial values of *R*_*l,ik*_ are the same as *W*_*l,ki*_ then equality will hold throughout the update iterations resulting in a symmetric system performing precise back-propagation. This is the proposal in Zipser and Rumelhart (1990). We experiment with different initializations, so that the updates are not performing back-propagation, even in the long run after many iterations the weights are not equal, although their correlation increases. We show that classification rates remain very close to those obtained by back-propagation. In addition, in order to increase the plausibility of the model we also experiment with sparsifying the feedforward and feedback connections by randomly fixing half of each set of weights at 0.

**Remark 1:** It is important to note that the feedback activity δ_*l,i*_ replaces the feedforward activity *x*_*l,i*_ and needs to be computed before the update of the feedforward weights feeding into unit *i* and the feedback weights feeding out of that unit, but using the original *feedforward* activity *x*_*l*−1, *i*_ of the units in layer *l* − 1. This requires a very rigid sequencing of the algorithm from top to bottom.

**Remark 2:** The feedback signal propagates by computing a linear combination of the feedback signals in the higher layers, but is then multiplied by the term $\sigma^{\prime }\left(h_{l,i}\right)$. To simplify as much as possible we have employed a non-linearity σ of the form

(3) σ(h)=max(-1,min(1,h)),

which is simply a saturated linear function at thresholds −1 and 1, and σ′(*h*) = 1 if |*h*| ≤ 1 and 0 otherwise. Thus, the feedback activity δ_*l,i*_ is the linear combination of the feedback activities δ_*l*+1, *k*_ in the layer above unless

$$\begin{array}{l} |h_{l,i}|\geq 1,\text{or}|x_{l,i}|=1. \end{array}$$

i.e., bottom-up input *h*_*l,i*_ is too high or too low, in which case δ_*l,i*_ = 0. A local network to compute this thresholding is described in Appendix 1. The computation of the top level derivative $\delta_{L,i}=\partial L/\partial h_{L,i}$ will be discussed in the next section.

### 3.2. Loss Function

The softmax loss commonly used in deep learning defines the probability of each output class as a function of the activities *x*_*L,i*_ as follows:

$$\text{softmax}{\left(x_{L}\right)}_{c}=p_{c}=\frac{e^{x_{L,c}}}{\sum_{i=1}^{C}e^{x_{L,i}}},c=1,\ldots ,C.$$

The loss computes the negative log-likelihood of these probabilities:

$$L\left(x_{L},y\right)=-\overset{C}{\underset{i=1}{\sum }}x_{L,i}y_{i}+\log\overset{C}{\underset{i=1}{\sum }}e^{x_{L,i}},$$

where *y*_*c*_ = 1 if the class of the input is *c* and *y*_*i*_ = 0, *i* ≠ *c*. Thus, the initial feedback signal is:

$$\delta_{L,i}=\frac{\partial L\left(x_{L},y\right)}{\partial x_{L,i}}=y_{i}-p_{i}.$$

This requires the computation of the softmax function *p*_*i*_, which involves the activity of all other units, as well as exponentiations and ratios.

The classification loss function used here is motivated by the hinge loss used in standard linear SVMs. In the simplest case of a two class problem we code the two classes as a scalar *y* = ±1 and use only one output unit *x*_*L*_. Classification is based on the sign of *x*_*L*_. The loss is given by

$$\begin{array}{l} L\left(x_{L},y\right)=\max\left(1-x_{L}y,0\right). \end{array}$$

The derivative of this loss with respect to *x*_*L*_, is simply

$$\begin{array}{l} \frac{\partial L}{\partial x_{L}}=\{\begin{array}{ll} -y & \text{if }y\cdot x_{L}\leq 1\\ 0 & \text{otherwise}. \end{array} \end{array}$$

The idea is that the output *x*_*L*_ should have the same sign as *y* and be sufficiently large in magnitude. Once the output achieves that, there is no need to change it and the loss is zero.

Writing $x_{L}=W^{t}x_{L-1}$, this yields the perceptron learning rule *with margin* (see Shalev-Shwartz et al., 2011):

$$\begin{array}{l} \frac{\partial L}{\partial W_{i}}=\{\begin{array}{ll} -x_{L-1,i} & \text{if }y=1\text{ and }W^{t}x_{0}\leq 1\\ x_{L-1,i} & \text{if }y=-1\text{ and }W^{t}x_{L-1}\geq -1\\ 0 & \text{otherwise} \end{array}, \end{array}$$

If we think of the supervised signal as activating the output unit with δ_*L*_ = +1 for one class and δ_*L*_ = −1 for the other, unless the input is already of the correct sign and of magnitude greater than 1, then $\delta_{L}=-\partial L/\partial x_{L}$. The update rule can be rewritten as *W*_*i*_ ← *W*_*i*_ + η*ΔW*_*i*_ where Δ*W*_*i*_ = δ_*L*_ · *x*_*L*−1,*i*_ if $x_{L}=W^{t}x_{L-1}$ satisfies δ_*L*_*x*_*L*_ ≤ 1. In other words if the output *x*_*L*_ has the correct sign by more than the margin of **1** then no update occurs, otherwise the weight is updated by the product of the target unit activity and the input unit activity. In that sense the update rule is Hebbian, except for shut down of the update when *x*_*L*_ is “sufficiently correct”.

One might ask why not use the unconstrained Hebbian update Δ*W*_*i*_ = ηδ_*L*_*x*_*L*−1, *i*_, which corresponds to a loss that computes the inner product of *y* and *x*. Unconstrained maximization of the inner product can yield over fitting in the presence of particularly large values of some of the coordinates of *x* and create an imbalance between the two classes if their input feature distribution is very different. This becomes all the more important with multiple classes, which we discuss next.

For multiple classes we generalize hinge loss as follows. Assume as before *C* output units *x*_*L*, 1_, …, *x*_*L,C*_. For an example *x*, of class *c* define the loss

(4) $$\begin{array}{l} L\left(x_{L},y\right)=\max\left(1-x_{L,c},0\right)+\mu \underset{i\neq c}{\sum }\max\left(1+x_{L,i},0\right). \end{array}$$

where μ is some balancing factor. The derivative has the form:

(5) $$\begin{array}{l} \frac{\partial L(x_{L},y)}{\partial x_{L,i}}=\{\begin{array}{ll} -1 & \text{ if }i=c\text{ and }x_{L,i}\leq 1\\ \mu & \text{ if }i\neq c\text{ and }x_{L,i}\geq -1\\ 0 & \text{ otherwise}. \end{array} \end{array}$$

Henceforth we will set $\delta_{L,i}=-\partial L\left(x_{L},y\right)/\partial x_{L,i}.$ Substituting the feedback signal δ_*L,i*_ for the feedforward signal *x*_*L,i*_ at the top layer has the following simple form:

(6) $$\begin{array}{l} \delta_{L,i}=\{\begin{array}{ll} 1 & \text{ if }i=c\text{ and }x_{L,i}\leq 1\\ -\mu & \text{ if }i\neq c\text{ and }x_{L,i}\geq -1\\ 0 & \text{ otherwise.} \end{array} \end{array}$$

and is then applied to compute the feedback to layer *L* − 1 - δ_*L*−1_ and the update of the weights *W*_*L*_, *R*_*L*_. All experiments below use this rule.

Note that δ_*L,i*_ is precisely the target signal, *except* when the feedforward signal has the correct value—greater than 1 if *i* = *c* (the correct class) and less than −μ for *i* ≠ *c* (the wrong class). This error signal only depends on the target value and input to unit *i*, no information is needed regarding the activity of other units. One can ask whether a neuron can produce such an output, which depends both on the exterior teaching signal and on the feedforward activity. In Appendix 1 we propose a local network that can perform this computation.

This loss produces the well-known one-vs.-all method for multi-class SVM's (see for example Hsu and Lin, 2002), where for each class *c* a two class SVM is trained for class *c* against all the rest lumped together as one class. Classification is based on the maximum output of the *C* classifiers. Each unit *x*_*L,c*_ can be viewed as a classifier of class *c* against all the rest. When an example of class *c* is presented it updates the weights to obtain a more positive output, when an example of any class other than *c* is presented it updates the weights to obtain a more negative output. Other global multiclass losses for SVM's can be found in Hsu and Lin (2002). In Amit and Mascaro (2003) and Amit and Walker (2012) a network of binary neurons with discrete synapses was described that implements this learning rule to update connections between discrete neurons in the input and output layers and with positive synapses. Each class was represented by multiple neurons in the output layer. Thus, classification was achieved through recurrent dynamics in the output layer, where the class with most activated units maintained sustained activity, whereas activity in the units corresponding to other classes died out.

## 4. Experiments

**Figure 3:**
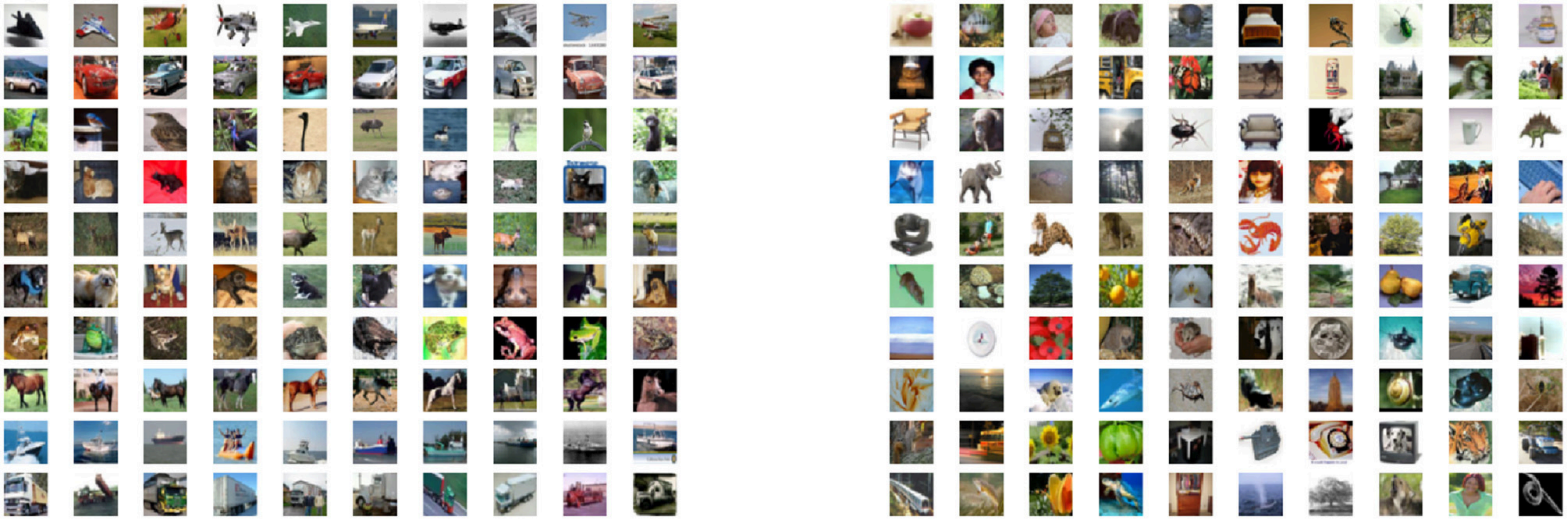
**(Left)** Each row showing 10 images from one of the 10 cifar10 classes. **(Right)** One image from each of the 100 classes in cifar100.

We report a number of experiments comparing the updated (URFB) to the fixed feedback matrix (FRFB) and comparing the multi-class hinge loss function to the cross-entropy with softmax loss. We restrict ourselves to image data. Since it is quite easy to obtain good results with the widely used MNIST handwritten data set (LeCun et al., 1999) we focus on two more challenging data sets called CIFAR10 and CIFAR100 (Krizhevsky et al., 2013). Each dataset contains 32x32 color images from 10 classes for the first and 100 classes for the second. The classes are broadly defined so that the category bird will contain multiple bird types at many different angles and scales. Some sample images are shown in Figure 3. Each data set has 50,000 training images and 10,000 test images.

There are a number of benchmark network architectures that have been developed over the past decade with good results on these datasets, see (Krizhevsky et al., 2012; Simonyan and Zisserman, 2014; Kaiming et al., 2016). These networks are very deep and employ a variety of methods to accelerate convergence, such as adaptive time-steps and batch normalization. These improvements involve steps that are not easily modeled as neural computations. For that reason we restrict our learning method to the simplest form of gradient descent with a fixed time step and no normalization. We do not perform any pre-processing of the input data, nor do we employ any methods of data augmentation to improve classification results. All our weights are initialized based on the method described in Glorot and Bengio (2010). Weights are uniformly drawn between [−*b*_*l*_, *b*_*l*_] where *b*_*l*_ is a function of the number of incoming and outgoing connections to a unit in layer *l*.

In the experiments we demonstrate the following:

1. With regular back-propagation (BP) hinge loss performs slightly worse than the softmax loss but results are comparable.
2. For shallow networks URFB performs somewhat better then FRFB but mainly converges faster. It never performs as well as BP but is close.
3. For deeper networks URFB again performs close to BP but FRFB performance degrades significantly.
4. With locally connected—untied—layers replacing convolutional layers results are slightly worse overall but the relationship between the different methods is maintained.
5. In URFB the feedback weights are never the same as the feedforward weights, although the correlation between the two sets of weights increases as a function iteration.
6. Even in initial iterations, when the weights are far from being aligned, training, and validation error rates decrease at similar rates to back propagation.

We first experiment with a shallow network with only two hidden layers, one convolutional and one fully connected.


simpnet: Conv 32 5x5; Maxpool 3; Drop .8;
           Full 500; Drop .3; Output


The notation Conv 32 5x5 means that we have 32—5x5 filters, each applied as a convolution to the input images, producing 32 output arrays of dimension 32x32. Maxpool 3 means that at each pixel the maximal value in the 3x3 window centered at that pixel is substituted for the original value (padding with 0's outside the grid), in each of the 32 output arrays, and then only every second pixel is recorded producing 32 arrays of size 16x16. Drop 0.8 means that for each training batch, a random subset of 80% of the pixels in each array are set to 0 so that no update occurs to the outgoing synaptic weights. This step was introduced in Srivastava et al. (2014) as a way to avoid overfitting to the training set. It is also attractive as a model for biological learning as clearly not all synapses will update at each iteration. Full 500 means a layer with 500 units, each connected to every unit in the previous layer.

The Output layer has *C* output units one for each class. We use the saturated linearity σ(*x*) = min(max(*x*, −1), 1) and the hinge loss function as given in (4). The update is a simple SGD with a fixed time step of 0.1, and the network is trained for 1,000 epochs with batch-size of 500. We make a point to avoid any adaptive normalization layers as these require a complex gradient that is not amenable to simple neural computations. We avoid the more sophisticated time step adaptations which depend on previous updates and some normalizations, which again do not seem amenable to simple neural computations.

The three parameters we adjusted were the time step and two drop out rates. We experimented with time-steps 0.01, 0.1, and 1.0 for the simpnet and found the best behavior on a held out validation set of 5,000 samples was with the value 0.1. We kept this value for all further experiments. We had two dropout layers in each network. One between convolutional layers and one before the output layer. The values were adjusted by running a few tens of iterations and making sure the validation loss was closely tracking the training loss.

**Figure 4:**
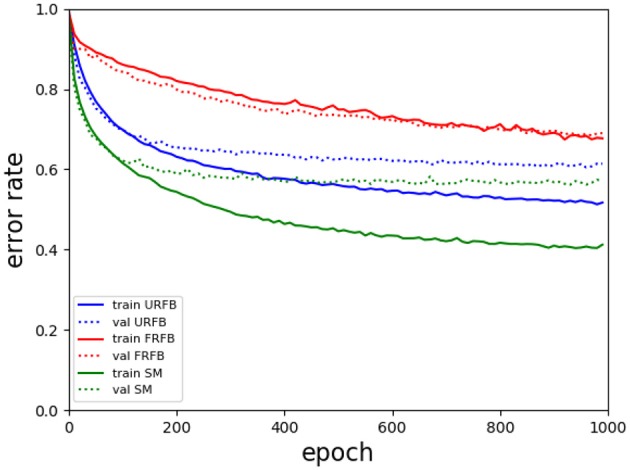
Evolution of error rates for simpnet as a function of epochs. Solid lines training error, dotted lines validation error. Green–BP, Blue–URFB, Red–FRFB.

**Figure 5:**
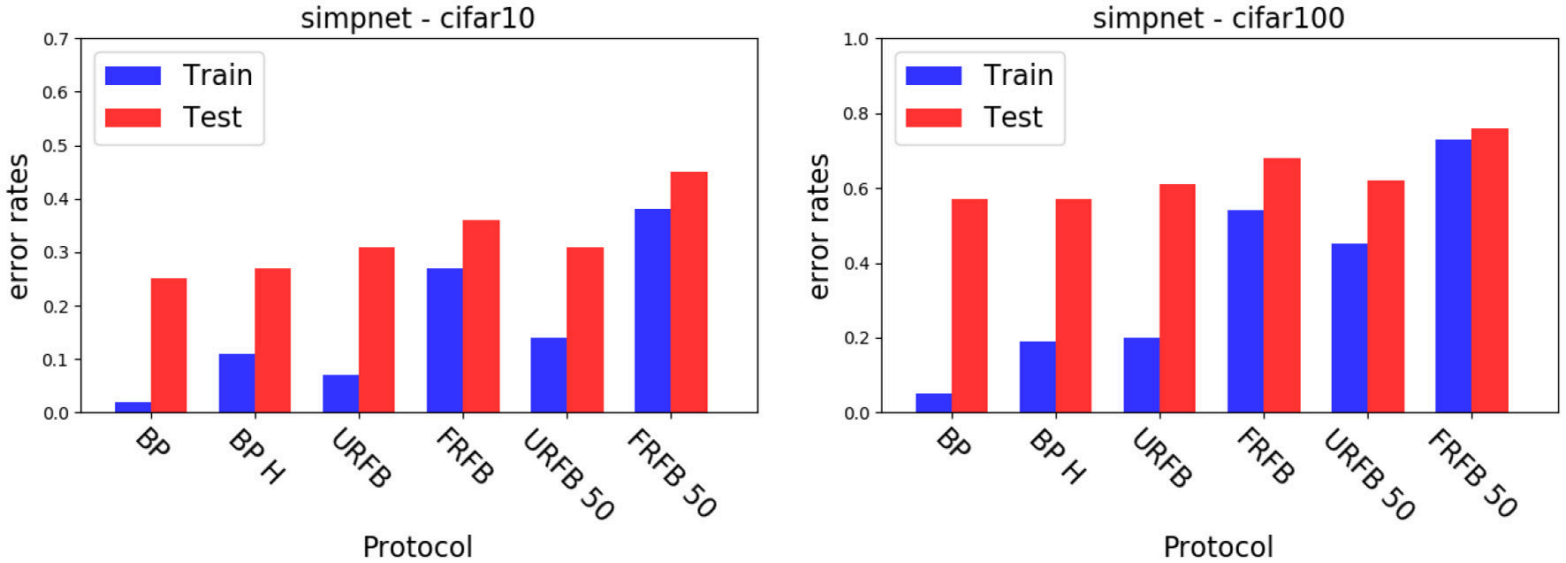
Error rates for simple network with different update protocols and different losses. **(Left)** CIFAR10, **(Right)** CIFAR100. BP, back-propagation with softmax and cross entropy loss; BP-H, back propagation with hinge loss, all other protocols use the hinge loss as well; URFB, Updated random feedback; FRFB, Fixed random feedback. 50% refers to random connectivity.

We also experiment with pruning the forward and backward connections randomly by 50%. In other words half of these connections are randomly set to 0. The evolution of error rates for the different protocols for simpnet as a function of protocol can be seen in Figure 4. Error rates for CIFAR10 and CIFAR100 datasets are shown in Figure 5. We note that the use of the multi-class hinge loss leads to only a small loss in accuracy relative to softmax. All experiments with random feedback are performed with the hinge loss. For CIFAR10 the difference between R fixed - FRFB - and R updated - URFB - is small, but becomes more significant when connectivity is reduced to 50% and with the CIFAR100 database.

Note that in the simple network the only layer propagating back an error signal is the fully connected layer. The first layer, which is convolutional, does not need to back-propagate an error.

We experiment with a deep network with multiple convolutional layers, and observe an even larger difference between R fixed and R updated. With the deep network FRFB performs very poorly. The deep architecture is given here.


deepnet: Conv 32 5x5; Maxpool 3; Conv 32
           3x3; Conv 32 3x3; Maxpool 3;
           Drop .8;
           Conv 32 3x3; Conv 32 3x3; Maxpool
           3; Drop .3; Full 500; Output


Finally we try an even deeper network with residual layers as in Kaiming et al. (2016). This means that after every pair of consecutive convolutional layers at the same resolution we introduce a layer that adds the two previous layers with no trainable parameters. This architecture was found to yield improved results on a variety of datasets.


deepernet: conv 16 3x3; conv 16 3x3;
             SUM; conv 32 3x3; conv 32 3x3;
             SUM; maxpool 3; drop .5; conv
             64 3x3; conv 64 3x3; SUM;
             maxpool 3; conv 128 3x3; conv 128
             3x3; SUM; maxpool 3; drop .8;
             full conn. 500; output


**Figure 6:**
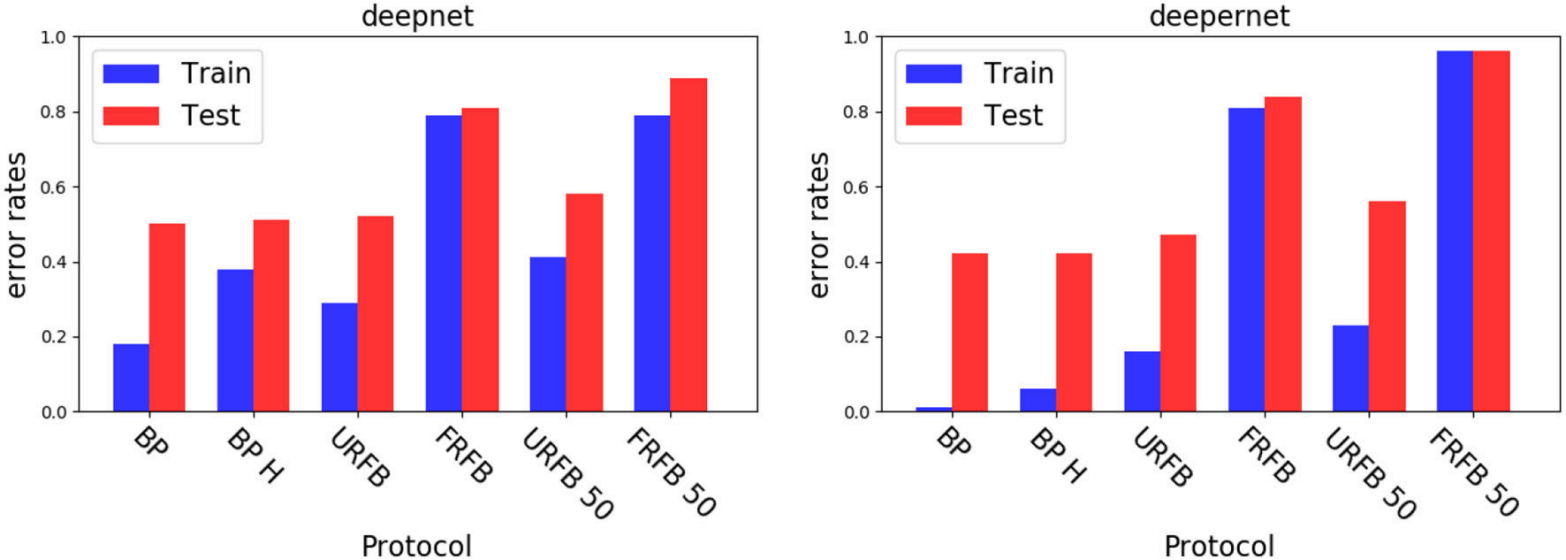
Error rates for the deepnet **(Left)** and deepernet **(Right)**. BP, back-propagation with softmax and cross entropy loss; BP-H, back propagation with hinge loss, all other protocols use the hinge loss as well; URFB, Updated random feedback; FRFB, Fixed random feedback. 50% refers to random connectivity.

**Figure 7:**
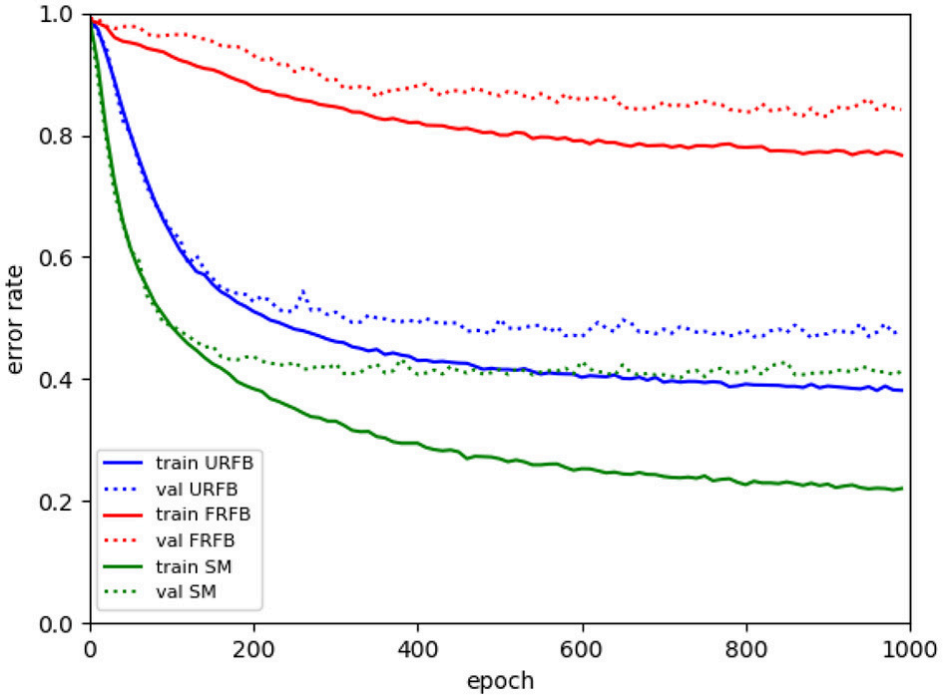
Evolution of error rates for deepernet as a function of epochs. Solid lines training error, dotted lines validation error. Green–BP, Blue–URFB, Red–FRFB.

We see in Figure 6 that for the default BP with softmax or hinge loss the error rate decreases from 50% with deepnet to 42% with deepernet. URFB also shows a decrease in error between deepnet and deepernet and again FRFB performs very poorly. The evolution of error rates for the different protocols as a function of iteration can be seen in Figure 7.

### 4.1. Untying the Convolutional Layers - Locally Connected Layers

We explore “untied” local connectivities determined by the corresponding convolutional layer. These are also called locally connected layers (Bartunov et al., 2018). A convolution corresponds to multiplication by a sparse matrix where the entry values are repeated in each row, but with some displacement. This again is not plausible because it assumes identical weights across a retinotopic layer. Furthermore the back-propagation update of a particular weight in a convolutional layer computes the *sum* of all products $\underset{i}{\sum }\delta_{l,i}x_{l-1,i+k}$, where *i* represents locations on the grid and *k* is a fixed displacement. So, it assumes that each one of the identical weights is updated by information summed across the entire layer.

**Figure 9:**
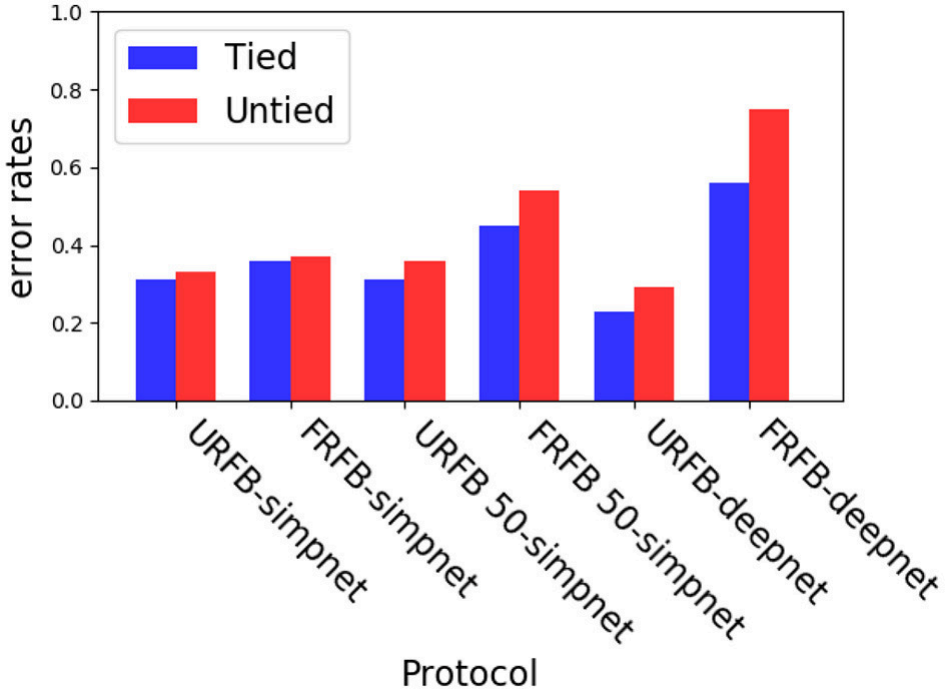
Experiments with untying the convolutional layers on simpnet and deepnet_s. Blue–convolutional layers (tied), Red–untied.

To avoid these issues with biological plausibility we instead assume each of the entries of the sparse matrix is updated separately with the corresponding product δ_*l,i*_*x*_*l*−1, *i*+*k*_. Only non-zero elements of the sparse matrix, that correspond to connections implied by the convolutional operation are updated. This is implemented using tensorflow sparse tensor operations, and is significantly slower and requires more memory than the ordinary convolutional layers. The error rates are similar to those with the original convolutional layers even with the deeper networks. In Figure 9 for CIFAR10, we show a comparison of error rates between networks with convolutional layers to networks with corresponding untied layers for the different training protocols. We show comparisons for simpnet and deepnet_s defined below.

**Figure 8:**
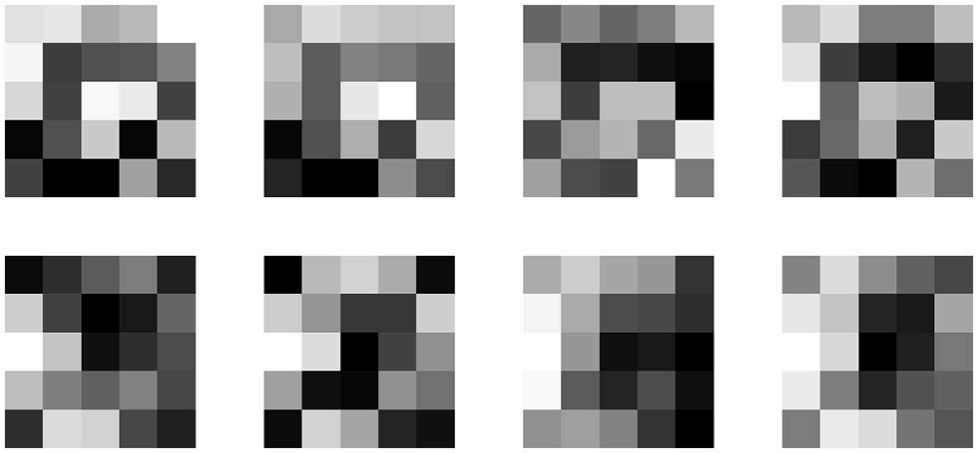
Corresponding filters extracted from the sparse connectivity matrix at four different locations on the 32x32 grid. Each row corresponds to a different filter.

Despite the fact that the weights are updated without being tied across space, the final connectivity matrix retains a strong spatial homogeneity. In other words at each location of the output layer one can restructure the weights to a filter and inspect how similar these filters are across locations. We presume that this is due to the fact that in the data local structures are consistent across space. In Figure 8 we show a couple of these 5x5 filters across four different locations in the 32x32 grid in the trained simpnet. We see that even after 1,000 iterations there is significant similarity in the structure of the filters despite the fact that they were updated independently for each location.

We also experiment with a deeper network:


deepnet_s: conv 16 3x3; conv 16 3x3;
  SUM;maxpool 3, stride 3; drop .5;
          conv 64 3x3; conv 64 3x3; SUM;
          maxpool 2, stride 2;
          conv 64 2x2; conv 64 2x2; SUM;
          maxpool 2, stride 2; drop .5;
          full conn. 500; output


Here we could not run all convolutional layers as untied layers due to memory constraints on our GPUs. Instead we ran the network for 100 epochs with the regular convolutional layers, then we froze the first layer and retrained the remaining layers from scratch using the untied architecture, see Figure 9. This would mimic a situation where the first convolutional layer perhaps corresponding to V1 has connections that are predetermined and not subject to synaptic modifications. Once more, we see that the untied layers with URFB reach error rates similar to those of the regular convolutional layers with standard gradient descent. And again, we observe that with a deeper network FRFB performance is much worse.

### 4.2. Weight Alignment

**Figure 10:**
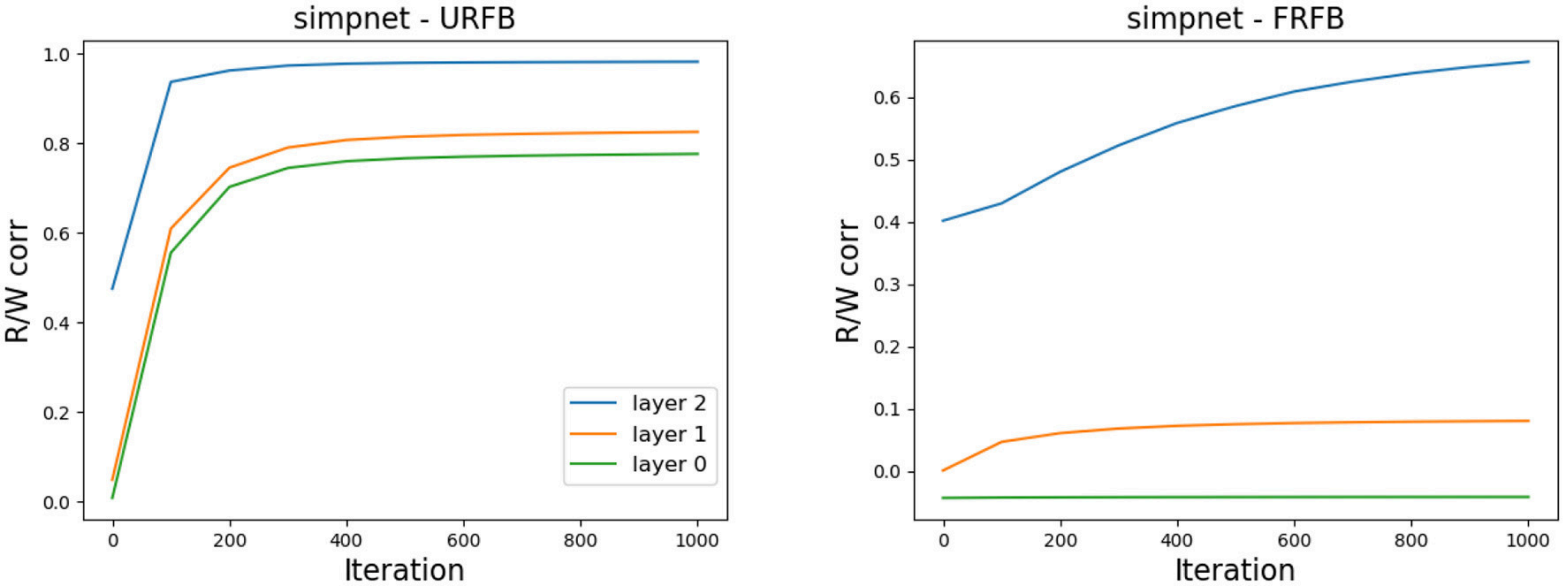
Correlation between *W*_*l*_ and *R*_*l*_ for the three layers in simpnet. **(Left)** URFB, **(Right)** FRFB.

One of the claims in Lillicrap et al. (2016) is that the network gradually aligns the updated feedforward weights to the fixed feedback weights. In Figure 10 we show the evolution of the correlations between the feedforward weights *W*_*l*_ and *R*_*l*_ for simpnet. Recall that the layer with highest index is the output layer and typically reaches high correlations in both URFB and FRFB. We see, however, that the alignment is much stronger for the URFB. Note that when weights are highly correlated the network is effectively implementing back-propagation.

**Figure 11:**
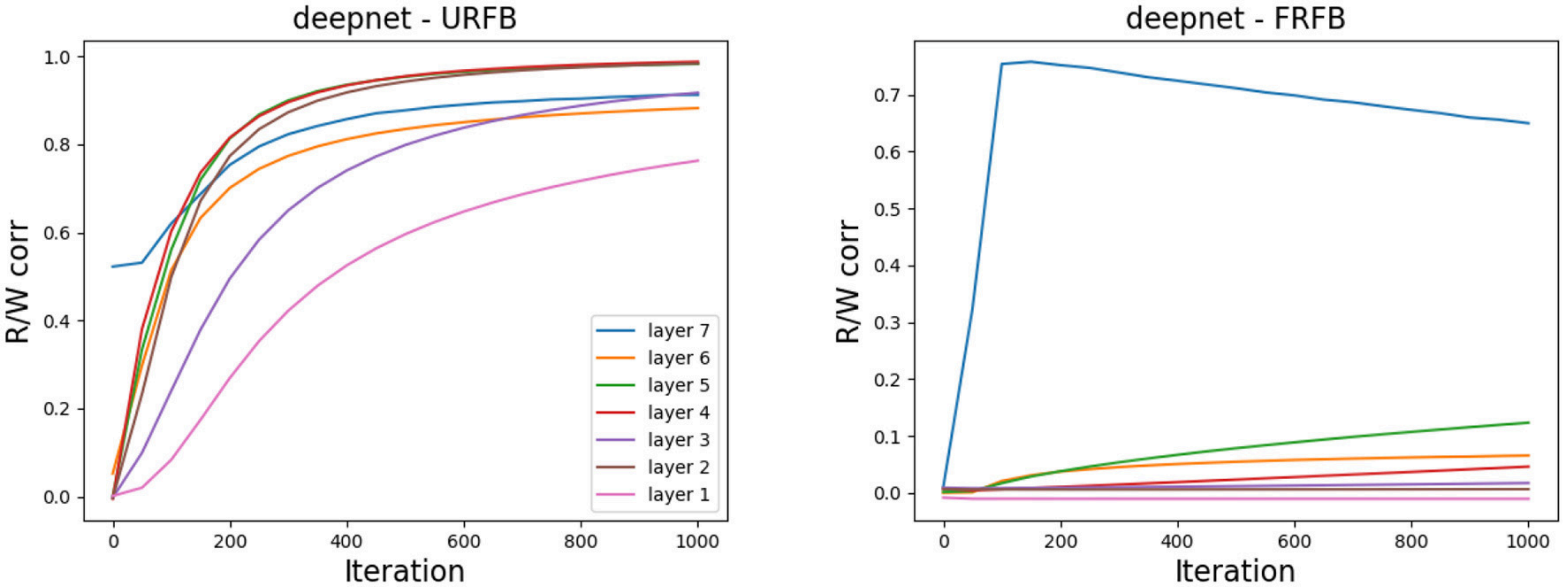
Correlation between *W*_*l*_ and *R*_*l*_ for the seven updated layers in deepnet_s. **(Left)** URFB, **(Right)** FRFB.

In Figure 11 we again show the evolution of the correlations between *W*_*l*_, *R*_*l*_ for the seven updated layers of the deeper network deepnet_s. Note that for some but not all layers the final correlations are very close to one. However, the training loss and error rates change very rapidly in the initial iterations when the correlations are very low. Interestingly the correlation levels are not a monotone function of layer depth.

## 5. Mathematical Analysis of Updated Random Feedback

The mathematical analysis closely follows the methods developed in Saxe et al. (2013) and thus focuses on linear networks, i.e., σ(*x*) = *x* and a simple quadratic loss. We start with a simple two layer network.

Let the input $x\in {\mathbb{R}}^{n_{0}}$, and the output $y=W_{2}W_{1}x\in {\mathbb{R}}^{n_{2}}$ with weights $W_{1}\in {\mathbb{R}}^{n_{1}\times n_{0}},W_{2}\in {\mathbb{R}}^{n_{2}\times n_{1}}$. If *X* is the *n*_0_ × *N* matrix of input data and *Y* the *n*_2_ × *N* of output data the goal is to minimize

$$C\left(W_{1},W_{2}\right)=|Y-W_{2}W_{1}X{|}^{2}.$$

We write $T=YX^{t}\in {\mathbb{R}}^{n_{2}\times n_{0}}$, and assume that *XX*^*t*^ = *I*, namely the input coordinates are uncorrelated. The gradient of *L* with respect to *W*_1_ and *W*_2_ yields the following gradient descent ODE's, which corresponds to regular back-propagation:

$$\begin{array}{l} \dot{W_{2}}=(T-W_{2}W_{1})W_{1}^{t}\\ \dot{W_{1}}=W_{2}^{t}(T-W_{2}W_{1}), \end{array}$$

with some initial condition *W*_1_(0), *W*_2_(0). If we implement the FRFB or URFB described above we get the following three equations:

(7) $$\begin{array}{l} \dot{W_{2}}=(T-W_{2}W_{1})W_{1}^{t}\\ \dot{W_{1}}=R_{2}(T-W_{2}W_{1})\\ \dot{R_{2}}=\epsilon W_{1}{\left(T-W_{2}W_{1}\right)}^{t}, \end{array}$$

where $R2\in {\mathbb{R}}^{n_{1}\times n_{2}}$ and ϵ is a parameter. Setting ϵ = 0 corresponds to FRFB, as there is no modification of the matrix *R*. The URFB corresponds to ϵ = 1. Our goal is to show that the larger ϵ the faster the convergence of the error to 0.

To simplify the analysis of (7) we assume *W*_1_(0) = *W*_2_(0) = 0 and *R*_2_(0) is random. Then *R*2 = *R*_2_(0) + ϵ$W_{2}^{t}$ and the system reduces to

(8) $$\begin{array}{l} \dot{W_{2}}=(T-W_{2}W_{1})W_{1}^{t}\\ \dot{W_{1}}=(R_{2}(0)+\epsilon W_{2}^{t})(T-W_{2}W_{1}). \end{array}$$

For deeper networks, and again assuming the *W*_*l*_ matrices are initialized at 0, we have the following equations for URFB:

(9) $$\begin{array}{l} {Ẇ}_{k}=EW_{1}^{t}\cdots W_{k-1}^{t}\\ \vdots \\ {Ẇ}_{i}=\left(R_{i+1}\left(0\right)+\epsilon W_{i+1}^{t}\right)\cdots \left(R_{k}\left(0\right)+\epsilon W_{k}^{t}\right)EW_{1}^{t}\cdots W_{i-1}^{t}\\ \vdots \\ {Ẇ}_{1}=\left(R_{2}\left(0\right)+\epsilon W_{2}^{t}\right)\cdots \left(R_{k}\left(0\right)+\epsilon W_{k}^{t}\right)E, \end{array}$$

where $E=T-W_{k}\cdots W_{1},\;T\in {\mathbb{R}}^{n_{k}\times n_{0}}$ and $W_{i}\in {\mathbb{R}}^{n_{i}\times n_{i-1}},i=1,\ldots ,k$. Again our goal is to show that as ϵ increases from 0 to 1, the error given by *e* = *tr*(*E*^*t*^*E*) converges faster to 0.

The precise statements of the results and the proofs can be found in Appendix 2. Here we show through a simulation that convergence is indeed faster as ϵ increases from ϵ = 0 (FRFB) to ϵ = 1 (URFB).

### 5.1. Simulation

**Figure 12:**
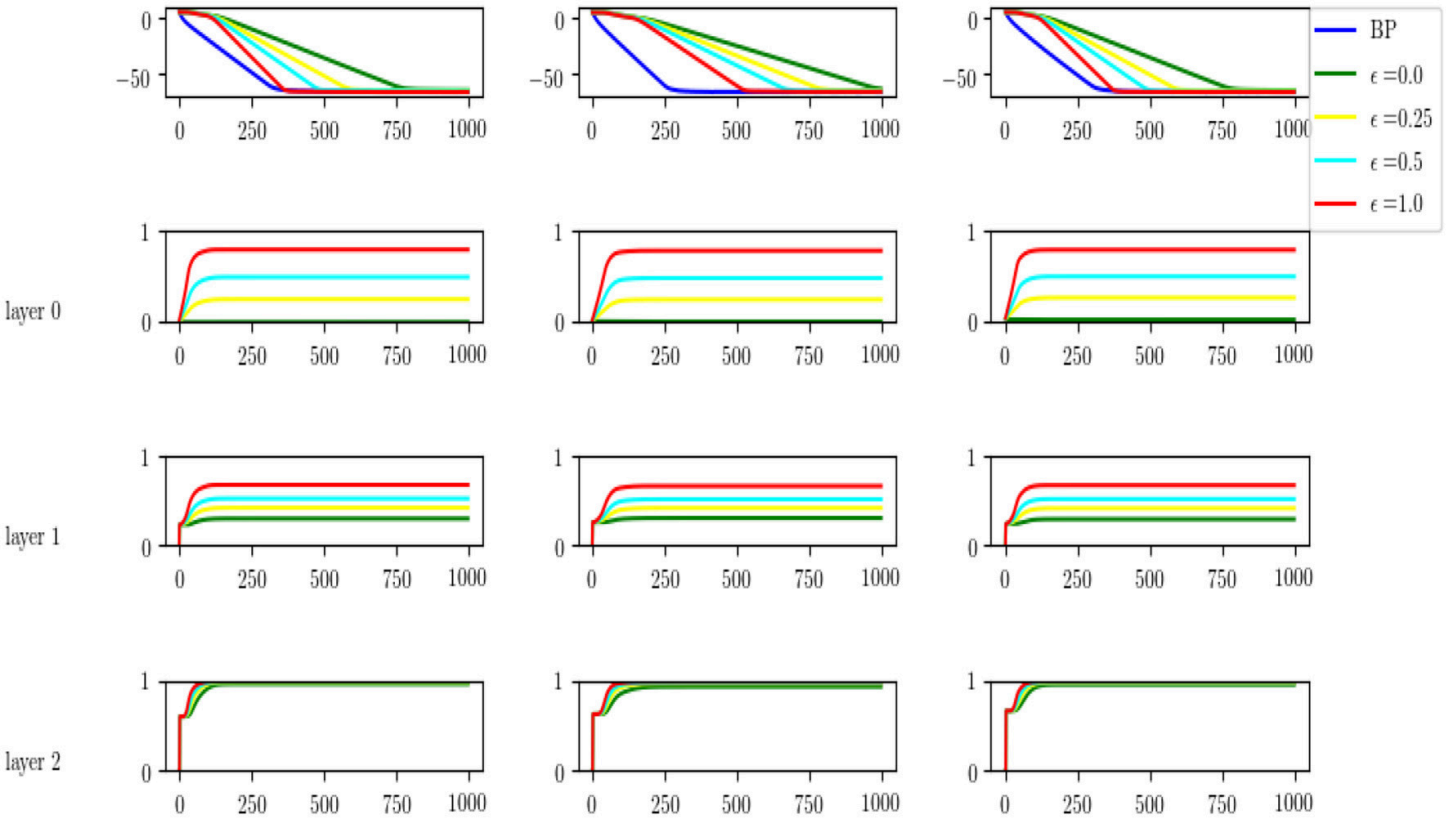
Top: comparison of log-error rates as a function of iteration for original BP and for four different values of ϵ = 0, 0.25, 0.5, 1. Results for three runs of the experiment. Last three rows, for each level of the network we show the evolution of the correlation between the *W* and *R*^*t*^ weights, for each of the values of ϵ.

We simulated the following setting. An input layer of dimension 40, two intermediate layers of dimension 100 and an output layer of dimension 10. We assume *X* = *I*_40_ so that $T=W_{1}^{*}W_{2}^{*}W_{3}^{*}$ with $W_{1}^{*}\in R^{40\times 100},W_{2}^{*}\in R^{100\times 100},W_{3}^{*}\in R^{100\times 10}.$ We choose the $W_{i}^{*}$ to have random independent normal entries with sd = 0.2. We then initialize the three matrices randomly as *W*_*i*_(0), *i* = 1, 2, 3 to run regular back propagation. For comparison we initialize *W*_*i*_(0) = 0 and initialize *R*_*i*_(0) randomly. We run the differential equations with ϵ = 0, 0.25, 0.5, 1., where ϵ = 0 corresponds to *FRFB* and ϵ = 1 to URFB. We run 1,000 iterations until all 5 algorithms have negligible error. We see the results in Figure 12. In the first row, for 3 different runs we show the log-error as a function of iteration, and clearly convergence rate increases with ϵ. In the three rows below that we show the evolution of the correlation of *W*_*l*_ and $R_{l}^{t}$ with the same color code. We see that for FRFB (green) the correlation of the weights feeding into the last layer increases to 1 but for the deeper layers that does not hold. Moreover, as ϵ increases to 1 the correlations approach higher values at each layer. The top layer always converges to a correlation very close to 1, lower layers do not reach correlation 1., and interestingly the correlation reached in the input layer is slightly higher than that of the middle layer. Similar non-monotonicity of the correlation was observed in the experiments in Figure 11.

## 6. Discussion

The original idea proposed in Zipser and Rumelhart (1990) of having separate feedback weights undergoing the same Hebbian updates as the feedforward weights yields the original back-propagation algorithm if the feedforward and feedback weights are initialized with the same values. We have shown that even when these weights are initialized differently the performance of the algorithm is comparable to that of back-propagation and significantly outperforms fixed feedback weights as proposed in Lillicrap et al. (2016). The improvement over fixed feedback weights increases with the depth of the network and is demonstrated on challenging benchmarks such as CIFAR10 and CIFAR100. We have also shown that in the long run the feedforward and feedback weights increase their alignment but the performance of the algorithm is comparable to back-propagation even at the initial iterations. We have introduced a cost function whose derivatives lead to local Hebbian updates and provided a proposal for how the associated error signal in the output layer could be implemented in a network. We have shown theoretically, in the linear setting, that adding the update to the feedback weights accelerates the convergence of the error to zero.

These contributions notwithstanding, there are still many aspects of this learning algorithm that are far from biologically plausible. First, although we have removed the need for symmetric connections, we have maintained a symmetric update rule, in that the update of a feedback and feedforward connection connecting two units is the same. To use the formulation in Gerstner et al. (2013) a typical Hebbian update has the form Δ*W* = *f*(*x*_*pre*_)*g*(*x*_*post*_), where *f, g* are typically *not* the same function, however in our setting both *f* and *g* are linear which yields a symmetric Hebbian update. In Burbank (2015) it is shown that a mirrored version of STDP could produce this type of symmetric update. Whether this is actually biologically realistic is still an open question.

Another important issue is the timing of the feedforward and feedback weight updates that needs to be very tightly controlled. The update of the feedforward and feedback connections between layer *l* and *l*+1 requires the feedback signal to layer *l*+1 to have replaced the feedforward signal in all its units, while the feedforward signal is maintained in layer *l*. This issue is discussed in detail in Guerguiev et al. (2017). They propose a neural model with several compartments. One that receives bottom-up or feedforward input and one that receives top-down feedback input. In a transient phase corresponding to the feedforward processing of the network the top-down input contribution to the neural voltage at the soma is suppressed. Then in a second phase this voltage is allowed in and combined with the feedforward voltage contribution to enable synaptic modifications. In our proposal, instead of combining the two voltages, the top-down voltage would replace the bottom up voltage. Still, in a multilayer network, this would need to be timed in such a way that the previous layer is still responding only to the feedforward input.

An important component of the model proposed in Roelfsema and Holtmaat (2018) are the synaptic tags that maintain the information on the firing of the pre and post-synaptic neurons allowing for a later synaptic modification based on some reinforcement signal. This may offer a mechanism for controlling the timing of the updates. An alternative direction of research would be to investigate the possibility of desynchronizing the updates, i.e., making the learning process more stochastic. If images of similar classes are shown in sequence it could be that it is not so important when the update occurs, as long as the statistics of the error signal and the feedforward signal are the same.

We have defined the network with neurons that have negative and positive values, and synapses with negative and positive weights. Handling negative weights can be achieved with properly adjusted inhibitory inputs. Handling the negative neural activity is more challenging and it would be of interest to explore an architecture that employs only positive neural activity. Finally we mention the issue of the training protocol. We assume randomly ordered presentation of data from all the classes, many hundreds of times. A more natural protocol would be to learn classes one at a time, perhaps occasionally refreshing the memory of previously learned ones. Because our loss function is local and updates to each class label are independent, one could potentially experiment with alternative protocols and see if they are able to yield similar error rates.

## Data Availability

Publicly available datasets were analyzed in this study. This data can be found here: a https://www.cs.toronto.edu/kriz/cifar.html.

## Code

Code for URFB can be found in https://github.com/yaliamit/URFB.git.

## Author Contributions

The author confirms being the sole contributor of this work and has approved it for publication.

### Conflict of Interest Statement

The author declares that the research was conducted in the absence of any commercial or financial relationships that could be construed as a potential conflict of interest.
