# Supplementary material for: Deep Learning With Asymmetric Connections and Hebbian Updates
Source: Front Comput Neurosci. 2019 Apr 4;13:18. doi: 10.3389/fncom.2019.00018 (PMC6458299; doi:10.3389/fncom.2019.00018)
Supplement: Supplementary file 1 [file Data_Sheet_1.pdf]

Appendix: Deep learning with asymmetric connections and Hebbian updates

Appendix 1: Networks for local thresholding

We describe two local networks, one for computing the error signal δ_L (see equation (6)) at the top level output units, and one to shut off the feedback signal $\delta_{l,i}$ based on (3).

Computing the output error signal

We first describe a network for computing the top level error signal δ_L, defined in equation (5), which depends on the activity of the output unit x_L as well as on the target signal. There have been many models proposed for Hebbian learning in terms of non-linear functions of both the presynaptic activity and the state of the post-stynaptic neuron, reviewed in detail in [**?**]. A model for the particular dependence required here, where synaptic modification stops when the input is sufficiently correct was proposed in [**?**]. A mechanism internal to the neuron is proposed, that shuts off potentiation or depression of its incoming synapses when the input is too high or too low. However, shutting off synaptic modifications, does not manifest itself in the activity of the neuron. An implementation that is internal to the neuron provides no explicit error signal, which can then be propagated to previous layers with the feedback connections. When the network only has two layers, an input and an output, that is not an issue, but with deeper networks we will need to use feedback connections to propagate the error signal.

as above, to avoid complications of modeling excitatory and inhibitory neurons we assume neurons have positive and negative firing rates and synaptic connections are positive and negative. The main idea is to introduce a control unit t_c that shuts the main unit o_c off when the input is outside the appropriate range.

Let δ_c be the activity of a neuron associated with class c in the top layer, with input given by $h_c = \langle W_c, x \rangle$. For simplicity we omit the index of the input layer. Assume h_c always lies in the interval $[-M, M]$. Given a learning threshold S (in the previous section we had $S = 1$), we want $\delta_c = 1$ if class c is presented and $h_c < S$, $\delta_c = -\mu$ if another class is presented and $h_c > -1$ and $\delta_c = 0$ otherwise. Let s_c be the unit providing the supervisory signal: 1 if class c is being presented, -1 otherwise, and let t_c be a 'control' neuron. The

input to t_c is simply o_c and the full input of o_c is

$$H_c = h_c + 2Ms_c - 2Mt_c,$$

Also $\delta_c = \sigma_\delta(H_c), \quad t_c = \sigma_t(o_c)$.

Let $\mu \leq 1$. The transfer functions of δ_c, t_c are given by

$$\sigma_\delta(x) = \begin{cases} 1+\epsilon & \text{if } x > 2M+S \\ 1 & \text{if } x \in [M, 2M+S] \\ 0 & \text{if } x \in [-M, M] \\ -\mu & \text{if } x \in [-2M-S, -M] \\ -\mu-\epsilon & \text{if } x < -2M-S \end{cases}, \qquad \sigma_t(x) = \begin{cases} 1 & \text{if } x > 1 \\ 0 & \text{if } -\mu \leq x \leq 1 \\ -1 & \text{if } x < -\mu \end{cases}$$

and shown in figure 1.

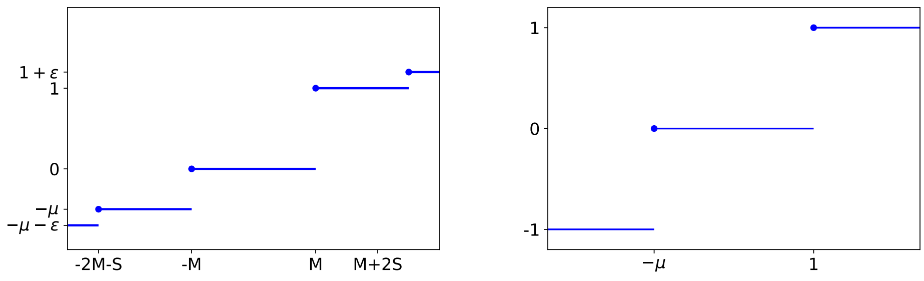

Figure 1: Left: Transfer function σ_v. Right: Transfer function σ_t.

If the supervisory signal is $s_c = 1$, t_c is initialized at 0 and $h_c \leq S$ then $M \leq H_c \leq 2M + S$, so $\delta_c = 1$ and $t_c = 0$. This yeilds the update cycle

$$\delta_c = 1 \to t_c = 0 \to \delta_c = 1,$$

and δ_c is constant at value 1.

If $h_c > S$ then $H_c > 2M + S$ so that $\delta_c = 1 + \epsilon$ and $t_c = 1$. Then, $H_c = h_c \in [-M, M]$

so that $\delta_c = 0$. This yields the update cycle:

$$\delta_c = 1 + \epsilon \to t_c = 1 \to \delta_c = 0 \to t_c = 0 \to \delta_c = 1 + \epsilon,$$

so that δ_c oscillates between 0 and $1 + \epsilon$.

Conversely if the supervisory signal is $s_c = -1$ and $h_c \geq -S$ then $-2M - S \leq H_c \leq -M$ so $\delta_c = -\mu$ and $t_c = 0$ yielding

$$\delta_c = -\mu \to t_c = 0 \to \delta_c = -\mu,$$

and δ_c is constant at $-\mu$. If $h_c < -S$ then $H_c < -2M - S$, $\delta_c = -\mu - \epsilon$ causing $t_c = -1$ and $H_c = h_c$ so that $\delta_c = 0$ and we get the update cycle:

$$\delta_c = -\mu - \epsilon \to t_c = -1 \to v_c = 0 \to t_c = 0 \to \delta_c = -\mu - \epsilon,$$

so that δ_c oscillates between 0 and $-\mu - \epsilon$.

In summary the activity of δ_c is input dependent. If $sign(\delta_c)h_c < S$ then $\delta_c = 1, -\mu$ depending on the presented class, and $\Delta W_{ic} = x_i \delta_c$. If $sign(\delta_c)h_c > S$ then δ_c oscillates and periodically visits the state $\delta_c = 0$ and no update of synapses connecting to δ_c occurs. Ignoring the oscillation this circuit can be thought of as implementing the rule in equation (6). The activity of δ_c is precisely the signal that needs to be propagated backwards to the input layer. This network is illustrated in figure 2.

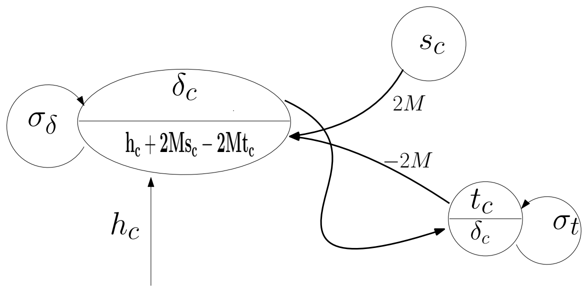

Figure 2: The circuit including the supervisory neuron s_c, the top layer neuron δ_c and the control neuron t_c.

Computing the shutdown of the feedback computation

We describe a local network to compute the expression in equation **??**. We add two control neurons $u_{l,i}, v_{l,i}$ with a simple threshold activation and input from $x_{l,i}$. We set $u_{l,i} = \mathbf{1}[\mathbf{x_{l,i}} \geq \mathbf{1}]$ and $v_{l,i} = -\mathbf{1}[\mathbf{x_{l,i}} \leq -\mathbf{1}]$. Only one of these units can be active. The input to unit l, i is the top-down feedback $\delta_{l,i}$, and we add $-2Ku_{l,i} + 2Kv_{l,i}$. Let

$$\sigma_\delta(u) = \begin{cases} u & \text{if } u \geq -K \\ 0 & \text{otherwise} \end{cases}$$

and set

$$\tilde{\delta}_{l,i} = \sigma_\delta(\delta_{l,i} - 2Ku_{l,i} + 2Kv_{l,i}).$$

Assuming $\delta_{l,i}$ is bounded between $-K, K$ then if either of the units $u_{l,i}, v_{l,i}$ are active $\delta_{l,i} - 2Ku_{l,i} + 2Kv_{l,i} \leq -K$ and $\tilde{\delta}_{l,i} = 0$, otherwise $\tilde{\delta}_{l,i} = \delta_{l,i}$. In terms of timing, before the feedback signal arrives at the unit, but after the value of $x_{l,i}$ is used to update the weights W_l, the original feedforward activity $x_{l,i}$ activates the units $u_{i,l}$ and $v_{i,l}$ so that their input is added to the incoming feedback signal $\delta_{i,k}$. This sequence of updates is illustrated in figure 3.

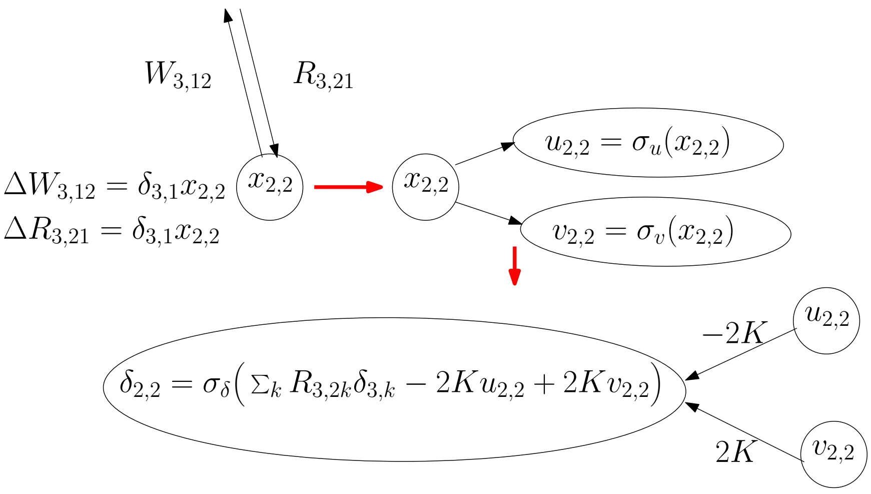

Figure 3: First weights connecting unit $x_{2,2}$ to layer above get updated based on the value of $x_{2,2}$. Second the activation of $u_{2,2}$ and $v_{2,2}$ is triggered. Third the computation of $\delta_{2,2}$ is performed in terms of $\delta_{3,k} and R_{3,2k}$ and set to 0 if either $u_{2,2}$ or $v_{2,2}$ are active.

Appendix 2: Statement of theorems and proofs

Let $T = U\Lambda_T V^t$ be the SVD of T. Assuming $n_k \leq n_0, n_1 \dots, n_{k-1}$ we have $U \in \mathbb{R}^{n_k \times n_k}, V \in \mathbb{R}^{n_0 \times n_k}$ and $\Lambda_T \in \mathbb{R}^{n_k \times n_k}$ positive diagonal. We set the initial condition for R_k by first picking n_k orthogonal vectors in $\mathbb{R}^{n_{k-1}}$ yielding an $n_{k-1} \times n_k$ matrix U_k and then writing $R_k(0) = U_k \Lambda_{R,k} U^t$ with $\Lambda_{R,k} \in \mathbb{R}^{n_k \times n_k}$ diagonal and positive and $U_k \in \mathbb{R}^{n_{k-1} \times n_k}$ with orthogonal columns. For general i choose $U_i \in \mathbb{R}^{n_{i-1} \times n_k}$ orthogonal, $\Lambda_{R,i} \in \mathbb{R}^{n_k \times n_k}$ positive and diagonal, and set $R_i(0) = U_i \Lambda_{R,i} U_{i+1}^t$. Write $W_i = U_{i+1} \Lambda_i U_i^t, i = 1 \dots, k$ and with $U_{k+1} = U$, and assume $\Lambda_i(0) = 0$ then the system in equation (9) in the main text decouples into n_k scalar equations one for each of the directions in U, V:

$$
\begin{aligned}
\dot{\lambda}_k &= e\lambda_{k-1} \cdot \lambda_1 \\
&\vdots \\
\dot{\lambda}_i &= (\lambda_{R,k} + \epsilon\lambda_k) \cdots (\lambda_{R,i+1} + \epsilon\lambda_{i+1}) e \lambda_{i-1} \cdots \lambda_1 \\
&\vdots \\
\dot{\lambda}_1 &= (\lambda_{R,k} + \epsilon\lambda_k) \cdots (\lambda_{R,2} + \epsilon\lambda_2) e,
\end{aligned}
\tag{1}
$$

with $\lambda_i(0) = 0$ and $\lambda_{R,i} > 0$ random. This implies that λ_i are increasing and e is always positive. Multiplying the i'th equation by $(\lambda_{R,i} + \epsilon\lambda_i)$ and the $i-1$'th equation by λ_{i-1} we get the equality

$$
\dot{\lambda}_i(\lambda_{R,i} + \epsilon\lambda_i) = \dot{\lambda}_{i-1}\lambda_{i-1}, i = 2, \dots, k.
$$

Since $\lambda_i(0) = 0$ we can integrate and get

$$
\lambda_{R,i}\lambda_i + \frac{\epsilon}{2}\lambda_i^2 = \frac{1}{2}\lambda_{i-1}^2 \tag{2}
$$

Rewriting $e = \lambda_T - \prod_{i=1}^k \lambda_i$, we have

$$
\begin{aligned}
\frac{\dot{e^2}}{2} = -e\sum_{i=1}^k \dot{\lambda}_i \prod_{j \neq i} \lambda_j &= -\, e^2 \sum_{i=1}^k \prod_{j=1}^{i-1} \lambda_j^2 \prod_{j=i+1}^k \lambda_j(\lambda_{R,j} + \epsilon\lambda_j) \\
&= -\, e^2 \sum_{i=1}^k \prod_{j=1}^{i-1} \lambda_j^2 \prod_{j=i+1}^k (\frac{\epsilon}{2}\lambda_j^2 + \frac{1}{2}\lambda_{j-1}^2)
\end{aligned}
\tag{3}
$$

where the third equality follows from (2). It follows that for each ϵ the error converges exponentially fast to 0 and if it can be shown that λ_i are increasing in ϵ the convergence rate is increasing with ϵ.

Two layer network

For a two layer network we have

$$\begin{aligned}\dot{\lambda}_2 =& (\lambda_T - \lambda_2\lambda_1)\lambda_1 \\ \dot{\lambda}_1 =& (\lambda_R + \epsilon\lambda_2)(\lambda_T - \lambda_2\lambda_1),\end{aligned} \tag{4}$$

assuming without loss of generality that $0 < \lambda_T \leq 1$ and $\lambda_R << \lambda_T$.

Theorem 1. *Define $e = \lambda_T - \lambda_2\lambda_1$, and assume $\lambda_1(0) = 0, \lambda_2(0) = 0$. Then e^2 converges exponentially fast to 0 and the rate of convergence increases as ϵ increases.*

Proof of Theorem 1. The differential equation fo the e^2 is given by:

$$\dot{e^2} = -2e^2(\lambda_1^2 + \frac{1}{2}\lambda_1^2 + \frac{\epsilon}{2}\lambda_2^2). \tag{5}$$

Since $\lambda_T, \lambda_R > 0$ and $\lambda_1(0) = \lambda_2(0) = 0$ we see that λ_1 and λ_2 are increasing in time, and so after a finite time are uniformly bounded away from 0. This implies that the error goes to 0 exponentially fast. We also note that $\lambda_1\lambda_2 = \lambda_T$ is a stationary point, so that $\lambda_1\lambda_2 \leq \lambda_T$ always holds. If we can show that the factor of $-e^2$ increases with ϵ then the rate of convergence of e to zero increases with ϵ.

Solving for λ_i in equation (2) and taking the positive solution we can write

$$\begin{aligned}\lambda_i =& G(\lambda_{i-1}, \lambda_{R,i}, \epsilon) \\ \lambda_{i-1} =& H(\lambda_i, \lambda_{R,i}, \epsilon),\end{aligned} \tag{6}$$

where

$$
\begin{aligned}
F(x, \lambda_R, \epsilon) =& \sqrt{\lambda_R^2 + \epsilon x^2}, \\
G(x, \lambda_R, \epsilon) =& \frac{F(x, \lambda_R, \epsilon) - \lambda_R}{\epsilon} = \frac{x^2}{F(x, \lambda_R, \epsilon) + \lambda_R}, \text{ and} \\
H(x, \lambda_R, \epsilon) =& \sqrt{2\lambda_R x + \epsilon x^2}.
\end{aligned} \tag{7}
$$

If we show that λ_1 increases with ϵ, then, by (6), $\sqrt{\epsilon}\lambda_2 = \sqrt{\lambda_R^2/\epsilon + \lambda_1^2} - \lambda_R/\sqrt{\epsilon}$, is easily seen to increase with ϵ since λ_1 and λ_R are positive. (In general, for any positive function $g(\epsilon)$ that is increasing in ϵ the function $f(\epsilon) = \sqrt{c^2/\epsilon + g(\epsilon)} - c/\sqrt{\epsilon}$ is increasing w.r.t to ϵ.) Thus $\epsilon\lambda_2^2$ is increasing in ϵ and the factor of $-e^2$ in equation (5) is increasing in ϵ.

Since $\lambda_2 = G(\lambda_1, \lambda_R, \epsilon)$ we get the scalar equation for λ_1 as

$$
\dot{\lambda}_1 = F(\lambda_1, \lambda_R, \epsilon)(\lambda_T - G(\lambda_1, \lambda_R, \epsilon)\lambda_1) \equiv f_1(\lambda_1, \epsilon) \tag{8}
$$

Let $h > 0$ be a small increment and write,

$$
\dot{\lambda}_\epsilon = f(\lambda_\epsilon, \epsilon), \quad \dot{\lambda}_{\epsilon+h} = f(\lambda_{\epsilon+h}, \epsilon + h).
$$

Denote $\Delta = \lambda_{\epsilon+h} - \lambda_\epsilon$ then to first order

$$
\dot{\Delta} = \frac{\partial f}{\partial \lambda}(\lambda_\epsilon, \epsilon)\Delta + \frac{\partial f}{\partial \epsilon}(\lambda_\epsilon, \epsilon).
$$

So if the second term is positive and the initial condition $\Delta(0) = 0$ then, using the formula for the solution to first order ODE's, Δ is always positive, so that λ_ϵ is increasing in ϵ. In our setting by (7)

$$
\begin{aligned}
\frac{\partial F(x, \lambda_R, \epsilon)}{\partial \epsilon} =& \frac{x^2}{2F(x, \lambda_R, \epsilon)} > 0, \\
\frac{\partial G(x, \lambda_R, \epsilon)}{\partial \epsilon} =& -\frac{x^4}{2(F(x, \lambda_R, \epsilon) + \lambda_R)^2 F(x, \lambda_R, \epsilon)} < 0,
\end{aligned}
$$

Consequently:

$$\frac{\partial f_1}{\partial \epsilon} = \frac{e\lambda_1^2}{2F(\lambda_1, \lambda_R, \epsilon)} + F\lambda_1 \frac{\lambda_1^4}{2(F(\lambda_1, \lambda_R, \epsilon) + \lambda_R)^2 F(\lambda_1, \lambda_R, \epsilon)} > 0.$$

Thus λ_1 is increasing in ϵ. □

Three layer network

Theorem 2. *For $k = 3$, assume $\lambda_1(0) = 0, \lambda_2(0) = 0$. Assume $\lambda_{R,2}, \lambda_{R,3} < \delta << \lambda_T < 1$, and assume $\lambda_{R,2} > \frac{1+\sqrt{1+\epsilon}}{2}\lambda_{R,3}$, then λ_1, λ_2 and λ_3 are increasing in ϵ so that e converges faster to 0 as ϵ increases.*

Proof of Theorem 2. With $k = 3$ equation (3) reduces to

$$\frac{\dot{e^2}}{2} = -e^2 \left[\left(\frac{\epsilon}{2}\lambda_2^2 + \frac{3}{2}\lambda_1^2 \right) \cdot \left(\frac{\epsilon}{2}\lambda_3^2 + \frac{1}{2}\lambda_2^2 \right) + \lambda_1^2\lambda_2^2 \right]. \tag{9}$$

We need to show that λ_1 and λ_2 are increasing in ϵ. The term $\epsilon\lambda_3^2$ can be handled just like the term $\epsilon\lambda_2^2$ in the case $k = 2$.

Write $H(\lambda_2) = H(\lambda_2, \lambda_{R,2}, \epsilon)$, $F(\lambda_2) = F(\lambda_2, \lambda_{R,3}, \epsilon), G(\lambda_2) = G(\lambda_2, \lambda_{R,3}, \epsilon)$ functions of λ_2. Furthermore set

$$\begin{aligned} G_2(\lambda_1) =& G(\lambda_1, \lambda_{R,2}, \epsilon), \\ G_3(\lambda_1) =& G(G_2(\lambda_1), \lambda_{R,3}, \epsilon), \\ F_2(\lambda_1) =& F(\lambda_1, \lambda_{R,2}, \epsilon), \\ F_3(\lambda_1) =& F(G_2(\lambda_1), \lambda_{R,3}, \epsilon) \end{aligned}$$

all functions of λ_1. Write the equations for λ_1, λ_2 with all other variables eliminated:

$$\begin{aligned} \dot{\lambda}_2 =& F(\lambda_2)\left(\lambda_T - G(\lambda_2)\lambda_2 H(\lambda_2)\right) H(\lambda_2) \\ \dot{\lambda}_1 =& F_3(\lambda_1)F_2(\lambda_1)\left(\lambda_T - G_3(\lambda_1)G_2(\lambda_1)\lambda_1\right) \end{aligned} \tag{10}$$

We have

$$\begin{aligned}
\frac{dF_2}{d\epsilon} =& \frac{\lambda_1^2}{2F_2} \\
\frac{dG_2}{d\epsilon} =& \frac{-\lambda_1^4}{2(F_2+\lambda_{R,2})^2 F_2} \\
\frac{dF_3}{d\epsilon} =& \frac{\partial F_3}{\partial G_2}\frac{\partial G_2}{\partial \epsilon} + \frac{\partial F_3}{\partial \epsilon} = \frac{G_2^2}{2F_3} - \frac{\epsilon G_2}{F_3}\frac{dG_2}{d\epsilon} \\
\frac{dG_3}{d\epsilon} =& -\frac{G_2^4}{2(F_3+\lambda_{R,3})^2 F_3} - \frac{\epsilon G_2}{F_3}\frac{\lambda_1^4}{2(F_2+\lambda_{R,2})^2 F_2} \\
\frac{dH}{d\epsilon} =& \frac{\lambda_2^2}{2H}
\end{aligned}$$

For λ_1 the derivative of the right hand side of (10) with respect to ϵ is

$$\frac{dF_3}{d\epsilon}F_2 e + F_3\frac{dF_2}{d\epsilon}e - F_3F_2\left[\frac{dG_3}{d\epsilon}G_2\lambda_1 + G_3\frac{dG_2}{d\epsilon}\lambda_1\right].$$

Since $F_2, G_2, F_3, G_3 > 0$ and the derivatives of G_2, G_3 with respect to ϵ are negative the second and third terms are positive. It is left to show that $\frac{dF_3}{d\epsilon} > 0$. Substituting $G_2 = \frac{\lambda_1^4}{(F_2+\lambda_{R,2})^2}$ in the first term we have:

$$\frac{G_2^2}{2F_3} - \frac{\epsilon G_2}{F_3}\frac{dG_2}{d\epsilon} = \frac{\lambda_1^4}{2F_3(F_2+\lambda_{R,2})^2}\left[1 - \frac{\epsilon G_2}{F_2}\right] = \frac{\lambda_1^4}{2F_3(F_2+\lambda_{R,2})^2}\frac{\lambda_{R,2}}{F_2} > 0.$$

For λ_2 the derivative of the right hand side of (10) with respect to ϵ:

$$\frac{\lambda_2^2}{2F}eH + F\left[\frac{\lambda_2^4}{2(F+\lambda_{R,3})^2 F}\lambda_2 H - G\frac{\lambda_2^2}{2H}\lambda_2\right]H + Fe\frac{\lambda_2^2}{2H} = T_1 + T_2 + T_3.$$

The first and third terms are positive. For the second term we have substituting $G = \frac{\lambda_2^2}{F+\lambda_{R,3}}$,

$$\begin{aligned}
T_2 = \left[\frac{\lambda_2^4}{2(F+\lambda_{R,3})^2 F}\lambda_2 H - G\frac{\lambda_2^2}{2H}\lambda_2\right] =& \frac{\lambda_2^5}{2}\left[\frac{H}{(F+\lambda_{R,3})^2 F} - \frac{1}{H(F+\lambda_{R,3})}\right] \\
=& \frac{\lambda_2^5}{(F+\lambda_{R,3})^2 FH}[H^2 - (F+\lambda_{R,3})F].
\end{aligned}$$

The last factor is

$$2\lambda_{R,2}\lambda_2 - \lambda_{R,3}^2 - \lambda_{R,3}\sqrt{\lambda_{R,3}^2 + \epsilon\lambda_2^2}.$$

Assume for small $\delta < 1$ that $\lambda_{R,2}, \lambda_{R,3} < \delta << \lambda_T$ then an order computation shows that as long as $\lambda_2 \leq \delta$ then $T_1 + T_3 = o(\delta^2)$ whereas $0 > T_2 = o(\delta^5)$ and so the sum is positive. When $\lambda_2 > \lambda_{R,2}, \lambda_{R,3}$, then the expression in (**??**) is bounded below by $2\lambda_{R,2}\lambda_2 - \lambda_{R,3}\lambda_2 - \lambda_{R,3}\sqrt{1+\epsilon}\lambda_2 > 0$ if $\lambda_{R,2} > \frac{1+\sqrt{1+\epsilon}}{2}\lambda_{R,3}$. Thus $T_2 > 0$. In summary both λ_1, λ_2 are increasing in ϵ as long as $\lambda_{R,2}, \lambda_{R,3} < \delta << \lambda_T$. □

References

[1] S. Fusi. Spike-driven synaptic plasticity for learning correlated patterns of mean firing rates. *Reviews in the Neurosciences*, 14:7384, 2003.

[2] Gerstner W., M. Lehmann, V. Liakoni, D. Corneil, and Brea J. Eligibility traces and plasticity on behavioral time scales: Experimental support of neohebbian three-factor learning rules. *Frontiers in Neural Circuits*, 12:53, 2018.
